# The community support and its role in adolescent pregnancy and school reintegration in rural Rwanda: a mixed-methods study

**DOI:** 10.1080/16549716.2024.2436716

**Published:** 2025-01-17

**Authors:** Fabien Nsanzabera, Evangeline Irakoze, Emmanuel Ntakirutimana, Alexis Manishimwe, Jean Bosco Nsengiyumva, Aimable Mwiseneza, Fabien Nkurikiyimana

**Affiliations:** Department of Education in Sciences, Faculty of Education, University of Technology and Arts of Byumba (UTAB), Byumba, Rwanda

**Keywords:** adolescent pregnancy, parental involvement, school dropout, reproductive health, socioeconomic factors

## Abstract

**Background:**

In low-income rural Rwanda, adolescent pregnancy limits health and education, leading to poor health outcomes, high dropout rates, and restricted socioeconomic mobility. While previous studies have inspected the prevalence, stigma, and health-related aspects of adolescent pregnancy in Rwanda, research is needed to investigate the impact of parental support and reproductive health education in these communities.

**Objectives:**

This research investigates the connection between adolescent pregnancy, socioeconomic status, and parental engagement in reproductive health education in rural Rwanda. It also assessed the availability of support resources and challenges faced by adolescent mothers in school.

**Method:**

A cross-sectional survey used structured questionnaires with 1,635 individuals across Nyanza, Bugesera, and Ngoma districts. Qualitative data from focus group discussions with adolescent mothers and community leaders explored educational obstacles and resource availability. Key informant interviews offered further insights. Descriptive and correlation analysis examined relationships between parental support, socioeconomic level, adolescent pregnancy, and school dropout.

**Results:**

Significant parental involvement in reproductive health education was reported by 68.3% of parents. Ninety-five percent of adolescent mothers reported that being pregnant had a detrimental impact on their academic achievement. Adolescent pregnancy was significantly associated with poor socioeconomic position, and correlation analysis verified this relationship with school dropout.

**Conclusion:**

The study highlights the importance of parental involvement and enhanced support services, such as reproductive health education and school-based adjustments, to reduce adolescent pregnancy and improve educational outcomes. These initiatives align with the Sustainable Development Goals, aiming to empower young mothers and reduce inequality.

## Paper Context

- **Main findings:** In Rwanda adolescent pregnancy strongly associated with parental involvement and socioeconomic factors, highlighting significant educational barriers faced by adolescent mothers.
- **Added knowledge:** This study underscores the critical importance of parental support and flexible educational programs on adolescent health and education in low-resource settings.
- **Global health impact for policy and action:** These findings support policy shifts toward inclusive reproductive health education and community support to enhance health and educational outcomes for adolescent mothers globally.

## Background

With broad social, economic, and health impacts, teenage pregnancy is still a major global public health concern, especially in low- and middle-income countries (LMICs) [1]. Approximately 12 million of the 21 million girls between the ages of 15 and 19 who become pregnant in LMICs give birth each year [2]. The frequency is significantly higher in sub-Saharan Africa, where 18% of young women give birth before turning 18 [3]. In Rwanda concerns regarding the fate of these young mothers are heightened by the rise in adolescent pregnancies [4]. Adolescent mothers frequently encounter obstacles to employment and education, which prolong poverty cycles and reduce their prospects for growth both on personal and professional levels [5]. The present situation poses substantial challenges to Rwanda’s advancements in reproductive health and gender equality, particularly considering Rwanda’s commitment to international frameworks like the convention on the elimination of all forms of discrimination against women, which advocates for women’s rights to education, health, and social protection [6]. Significant gaps exist, especially in rural regions, despite government efforts targeted at lowering adolescent pregnancies and promoting girls’ reintegration into the educational system [7].

It is commonly known that adolescent pregnancy has certain health hazards, such as increased rates of maternal death, preterm deliveries, and low birth weight [8]. The social stigma associated with pregnant adolescents increases these risks since it frequently results in social isolation, mental health issues, and troubles reintegrating into society [9]. In Rwanda, adolescent mothers are more likely to drop out of school, which would further limit their work opportunities in the future and widen social gaps. This is especially true in rural areas where cultural norms, restricted access to reproductive health care, and financial difficulties make these problems worse [10]. Even though the Rwandan government has implemented nationwide programs to reduce adolescent pregnancies, there are still many gaps in services, especially in the areas of education and family assistance [11]. Teenage pregnancies in rural areas are still on the rise despite these initiatives [4].

This study specifically addresses the research gap of identifying and evaluating the factors leading to adolescent pregnancy in rural areas and barriers adolescent mothers encounter when trying to reintegrate into the school system. By pinpointing the barriers experienced by adolescent mothers and assessing the role of family and community support, this study aims to address key obstacles to adolescent mothers’ socioeconomic advancement and educational continuity. The specific goals of this research were to: (1) determine the major causes of adolescent pregnancy in adolescent girls living in rural Rwanda; (2) evaluate the obstacles that adolescent mothers faced when reintegrating into school; and (3) assess the influence of family and community support on the reproductive health decisions and educational outcomes of adolescent mothers.

It has been shown that adolescent mothers’ support for going back to school is greatly influenced by cultural ideas and family attitudes, with many girls experiencing stigmatization that impedes their educational reintegration [12]. Similar results have been found internationally, with research showing that extensive family and community involvement is essential to enhancing reproductive health outcomes and young mothers’ educational possibilities [13]. The current study, which focuses on community dynamics and support networks, is in line with international initiatives to address adolescent pregnancy and education. It has global implications beyond Rwanda. Numerous LMICs face similar obstacles, such as limited access to reproductive health services, deeply rooted cultural norms, and economic limitations that compound the challenges faced by adolescent mothers [14]. The results offer valuable insights into how community and family dynamics influence the health and educational outcomes of adolescent mothers, and they may be used to develop intervention strategies in other similar contexts [15].

This study advances global efforts to meet the sustainable development goals (SDGs), particularly in the areas of health, education, and gender equality, by advancing a deeper understanding of the social and structural determinants influencing adolescent pregnancy and education in LMICs. In order to support adolescent mothers, it highlights the necessity of focused, culturally relevant interventions that involve the family and the community. These observations not only guide Rwanda’s domestic policies but also offer insightful guidance to other nations dealing with comparable issues, supporting global endeavours to accomplish sustainable and just development for all.

## Materials and methods

A cross-sectional descriptive study was carried out to assess the barriers experienced by adolescent mothers in Rwanda, perspectives of adolescent mothering, and factors leading to adolescent pregnancy. This study was conducted in three specific Rwandan districts: Bugesera, Ngoma, and Nyanza. These districts were chosen as representative settings because they demonstrate high incidences of adolescent pregnancy and limited resources for adolescent mothers. Bugesera, Ngoma, and Nyanza each have unique demographic and socio-economic factors, which contextualize the study’s findings within a broader framework of rural and resource-limited Rwandan communities. The target population’s opinions, experiences, and perceptions might be recorded at a specific moment in time attributable to this design.

**Figure 1.**
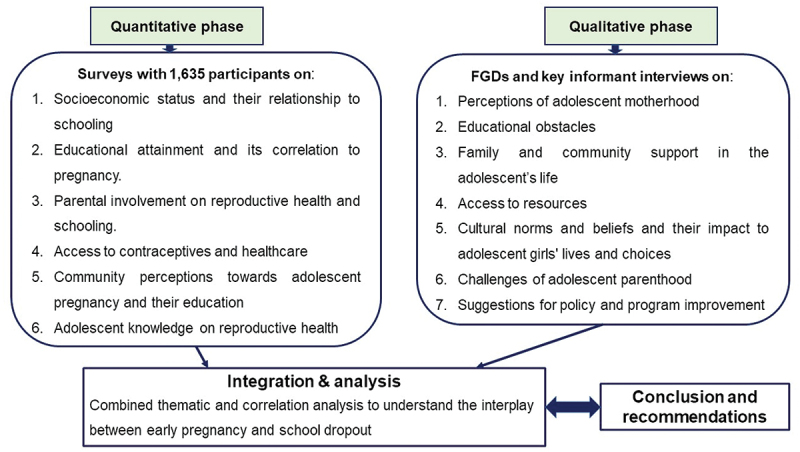
Flowchart of a concurrent mixed-methods study on adolescent motherhood.

The study design is described as concurrent mixed-methods. The two phases were conducted simultaneously with integration of quantitative and qualitative data in order to provide a comprehensive understanding of the issues. The quantitative component aimed to statistically assess factors contributing to adolescent pregnancy and its relationship with educational outcomes and socioeconomic variables, while the qualitative component explored in-depth perspectives of adolescent mothers and community views (Figure 1).

The study population included defined groups: adolescent mothers, community members, educators, parents, and healthcare providers. An ‘adolescent mother’ was defined as any female aged 10–19 years who had given birth. ‘Community people’ included local leaders, neighbors, and individuals with direct insight into community issues. ‘Educators’ referred specifically to primary and secondary school teachers, while ‘parents’ were defined as parents of adolescents who could provide insights into household and social influences. ‘Healthcare providers’ included midwives, nurses, and community health workers directly involved in adolescent health.

The study population comprised 225 participants from the Bugesera district who provided insights on community perceptions of education for adolescent mothers and 900 respondents from the Ngoma district who were surveyed to assess the factors contributing to adolescent pregnancy. Furthermore, 510 residents of the Nyanza area participated in a study aimed at evaluating parental involvement in reproductive health education.

The sample size was calculated based on a target confidence level of 95% and power of 80%, with adjustments for the population size of each district. Multistage sampling was employed to identify community people, educators, parents, healthcare providers, and teenagers as the potential study population. In the first phase, study sites were selected based on the high incidence of adolescent pregnancy and motherhood previously documented in the Rwanda Demographic and Health Survey 2014–15. The survey reported that the Eastern Province had the highest percentage of women aged 15–19 who had given birth, at 8.1% [16]. Stratified random sampling was used in the second stage of participant selection to guarantee representation from a range of age groups, educational backgrounds, and work situations. Finally, in order to concentrate on adolescent mothers who had experienced adolescent pregnancy and its effect on education, purposive sampling was employed. Inclusion criteria for the study were participants aged 10–19 for adolescent mothers and other groups who had a direct role in or perspective on adolescent health, while exclusion criteria included participants unwilling to provide informed consent or lacking involvement with adolescent health or education.

To provide thorough insights, both quantitative and qualitative data collection methods were used. The study employed structured questionnaires to gather information on demographics, contraception knowledge, adolescent pregnancy risk factors, and healthcare accessibility. These surveys included questions about sexual health education, family history, awareness of contraception, and the effect of adolescent pregnancy on education. Categorical response options were provided for these questions to capture the frequency of certain behaviors, attitudes, or perceptions. Focus group discussions (FGDs) with adolescent mothers and community leaders also explored their perspectives on educational obstacles, adolescent parenthood, and accessible resources. Each FGD consisted of 6–8 participants to ensure a manageable and interactive discussion. Key informant interviews with educators, healthcare professionals, and local leaders were also carried out in order to pinpoint institutional issues and suggest actions aimed at assisting adolescent mothers. To capture experiential depth, a phenomenological approach informed the qualitative components, focusing on the lived experiences of participants to contextualize adolescent mothering within community norms and structural challenges. The variables in the study were separated into independent and dependent groups. Outcome variables were educational dropout rates, attitudes on adolescent motherhood, and use of contraceptive services, while independent variables were factors like household income, access to family planning, and parental involvement. For questions about attitudes, categorical response options were used to measure responses; for questions about facts, binary alternatives were used.

Data collection was conducted from January to March 2024 across household settings, schools, and community centers using face-to-face interviews and self-administered questionnaires. Responses were recorded on tablets, and data were transferred to a central database daily to ensure accuracy. A trained research team facilitated all data collection, with sessions supervised by team leads to maintain consistency and quality. The SPSS software was used to analyse the data (version 19). Demographic characteristics, attitudes toward the use of contraceptives, level of education, and accessibility to healthcare were all described using descriptive statistics, such as frequencies and percentages. Correlation analysis was used to assess the relationship between adolescent pregnancy and school dropout rates. Qualitative data from focus group discussions and interviews were subjected to thematic analysis, which helped to discover important themes on stigma, difficulties in school, and societal perspectives on adolescent motherhood. We implemented techniques specific to each research component to guarantee the rigor of the study. Rich descriptions, a clear audit trail, and triangulated viewpoints improved the transferability and credibility of qualitative analysis. By validating survey instruments and evaluating internal consistency, quantitative rigor was attained while guaranteeing generalizability and reliability. A convergent parallel design made integration easier for this mixed-methods approach, which used triangulation to match quantitative patterns with qualitative themes. Our findings, supported by a systematic methodology, ensured reliable and valid results while offering actionable insights for addressing challenges in adolescent health and education in rural Rwanda. This evidence informs policies aimed at improving the lives and opportunities of adolescent mothers.

The ethics committee of the University of Technology and Arts of Byumba (UTAB) granted ethical clearance, and each participant provided informed consent. Adolescents under the age of 18 needed parental approval. Participation was entirely optional, and confidentiality was upheld. This approach offered a strong framework for investigating the complex problems of adolescents and adolescent pregnancy, guaranteeing that the results accurately represented the difficulties and realities faced by the community.

## Results

### Parental involvement, education, and peer influence on adolescent sexual health

We interviewed nine hundred (900) people from Ngoma district in Rwanda’s eastern province to assess various factors that contribute to adolescent pregnancy. The results of this study focus on adolescent pregnancy and reproductive health education in Rwanda, highlighting significant obstacles and opportunities for advancing health, well-being, and gender equality (Table 1). A substantial number of respondents indicated significant peer pressure regarding relationships and sexuality, with 93.3% of respondents reporting feeling pressured by peers and 95% seeing peer pressure related to early sexual activity or pregnancy. Additionally, 91.7% reported having their first sexual experience before turning 18, emphasizing the importance of thorough and early sexual education. According to qualitative findings from FGDs, adolescent mothers believed that peer pressure was everywhere; some mentioned that there were not enough role models encouraging healthy relationships.

**Table 1.** Adolescent survey on family structure, education, peer pressure, contraception.

Category	Subcategory	N	%
Family structure	Nuclear family	525	58.3
	Single-parent family	240	26.7
	Extended family	135	15.0
Parental involvement	Both parents actively involved	615	68.3
	One or no parent involved	285	31.7
Parental education	Secondary education	270	30.0
	Primary education	240	26.7
	No schooling	240	26.7
	Adult education/literate	150	16.7
Parental occupation	Unemployed	495	55.0
	Informal employment	150	16.7
	Full-time employment	90	10.0
	Self-employed	90	10.0
	Part-time employment	75	8.3
Peer influence	Felt pressure from peers	840	93.3
	No peer pressure	60	6.7
	Observed pressure related to early sexual activity or pregnancy	855	95.0
Sources of sexual health information	Sexual health clinics	870	96.7
	Schools	855	95.0
	Community health organizations	705	78.3
	Healthcare providers	600	66.7
	Online resources	165	18.3
Awareness and accessibility of contraception	Informed about contraception	705	78.3
	Not adequately informed	195	21.7
	Contraception easily accessible	600	66.7
	Contraception access difficult	300	33.3
Comfort in using reproductive health services	Very comfortable	390	43.3
	Somewhat comfortable	450	50.0
	Very uncomfortable	60	6.7
Age at first sexual encounter	Under 18	825	91.7
	Over 18	75	8.3
Contraceptive use at first encounter	Condom	480	53.3
	Emergency contraception	285	31.7
	Birth control pills	120	13.3
	Fertility awareness	15	1.7

N: Frequency, %: Percent; Source: This study.

Despite efforts like Rwanda’s adolescent sexual and reproductive health and rights policy [7], only 66.7% of respondents believed contraception techniques were freely accessible. However, 96.7% received sexual health information from clinics and 95% from schools. Although awareness is high, access remains a barrier that could reduce the effectiveness of educational initiatives. Regarding comfort in using reproductive health services, 50% of participants felt ‘somewhat comfortable’. Family dynamics also surfaced as a significant factor, with 58.3% of respondents coming from single-parent households and 26.7% from nuclear families. Notably, 31.7% reported a lack of active parental involvement, underscoring the need for family-inclusive approaches. Key informant interviews revealed that cultural beliefs frequently prevent parents from having an open conversation about reproductive health issues.

### Challenges, societal attitudes, and support needs of adolescent mothers

Two hundred and twenty-five (225) participants from Bugesera district shared their thoughts on adolescent pregnancy to assess the community’s awareness of adolescent motherhood (Table 2). Adolescent mothers in Rwanda face various concerns tied to broader social issues, aligning with sustainable development goals (SDGs) like SDG 1 (no poverty), SDG 3 (good health and well-being), and SDG 4 (quality education). All respondents agreed that poverty poses a significant obstacle, reflecting financial constraints affecting their health and education. Health risks are another critical concern; 86.7% of respondents cited the need for comprehensive healthcare services aligned with international health recommendations. The intersection of poverty and stigma was highlighted by qualitative data from FGDs, where participants described how adolescent mothers’ social isolation is frequently made worse by economic difficulties.

**Table 2.** Challenges, societal attitudes, and support for adolescent mothers in Rwanda.

Category	Subcategory	N	%
Challenges faced by adolescent mothers	Poverty	225	100.0
	Health risks	195	86.7
	Peer pressure	180	80.0
	Mental health problems	180	80.0
	Limited educational opportunities	165	73.3
	Inadequate knowledge of family planning	75	33.3
	Parental neglect	60	26.7
	Social constraints	45	20.0
Societal attitudes towards teen mothers	Irresponsible	195	86.7
	Inexperienced in parenthood	180	80.0
	Unethical	135	60.0
	Disengaged in productive roles	90	40.0
	Ignored by society	60	26.7
	Welfare dependency	60	26.7
	Labelled as impolite	30	13.3
	Seen as uninformed	30	13.3
	Considered uninteresting	15	6.7
Perceived importance of education	Very important	195	86.7
	Not important	30	13.3
Potential benefits of education for adolescent mothers	Promote their overall well-being	210	93.3
	Improving their financial stability	195	86.7
	Enhance their parenting abilities	165	73.3
	Better employment opportunities	90	40.0
Education system support for adolescent mothers	Yes	210	93.3
	No	15	6.7
Existing community programs	No	210	93.3
	Yes	15	6.7
Awareness of resources for adolescent mothers	Aware	150	66.7
	Doubtful if resources exist	60	26.7
	Don’t know	15	6.7
Necessary support for adolescent mothers	Childcare services	225	100.0
	Financial assistance	180	80.0
	Counseling and support groups	165	73.3
	Parenting education	90	40.0
	Mentorship programs	45	20.0
	Community resources	15	6.7

Source: This study.

Adolescent mothers face considerable societal stigma; attitudes toward them are marked by perceptions of immaturity (80%) and irresponsibility (86.7%), contributing to social exclusion and mental health challenges (80%). While 86.7% of respondents agreed on the importance of education, issues within the educational system persist. A majority (93.3%) of respondents think the system aids those who wish to return to school, but the absence of community programs (93.3% claimed none exist) highlights the lack of real-world support needed for educational access. Only 66.7% of respondents were aware of available support systems. Childcare (100%) and financial aid (80%) were identified as critical support services, and the emphasis on counseling and support groups (73.3%) elucidates the need for psychosocial support. Although Rwanda has initiatives like the national strategy for adolescent sexual and reproductive health, a lack of local programs indicates gaps in community-level support. Key informants further noted the inadequacy of current interventions in addressing the specific social and psychological needs of adolescent mothers.

### Impact of adolescent pregnancy on academic performance and education continuation

In Ngoma district, 70% of adolescent mothers stated that their decision to finish school was influenced by healthcare access. Table 3 shows that 98.3% of respondents reported that pregnancy negatively impacted their academic performance, with 98.3% missing school due to pregnancy-related issues, highlighting adolescent pregnancy’s adverse effects on education. Additionally, 80% of respondents cited school policies and assistance as essential factors for continuing education while pregnant, and healthcare access (70%) and financial resources (76.7%) also played significant roles. Furthermore, a correlation analysis was used to assess the relationship between adolescent pregnancy and school dropout rates. The results indicated a strong positive correlation (r = 0.78, p < 0.01), confirming a statistically significant association between pregnancy and increased dropout likelihood. Specifically, 90% of respondents considered dropping out of school due to pregnancy, with 26.7% expressing concerns about future opportunities. The need of empathy and inclusion was emphasized in focus group discussions with adolescent moms, who expressed feelings of despair about perceived prejudices in the school setting and the absence of specialized support programs.

**Table 3.** Academic impact and influential factors related to adolescent pregnancy.

Statement	Category	N	%
Impact of pregnancy on academic performance	Affected	885	98.3
	Not affected	15	1.7
Type of academic impact	Negative	885	98.3
School attendance due to pregnancy issues	Missed school	885	98.3
	No absences	15	1.7
Consideration of dropping out	Yes	810	90.0
	No	90	10.0
Influencing factors for continuing education	School policies and support	720	80.0
	Financial resources	690	76.7
	Access to healthcare	630	70.0
	Personal goals	630	70.0
	Future prospects	240	26.7
	Socio-economic factors	135	15.0
Awareness of consequences of adolescent Pregnancy	Aware	855	95.0
	Not aware	45	5.0
Correlation between adolescent pregnancy and school dropout rates	Pearson’s	*r* = 0.78	*p* < 0.01
	Data Points Used: Adolescent pregnancy rates and self-reported dropout intentions due to pregnancy	900	

Source: This study.

### Parental influence on adolescent reproductive health and adolescent pregnancy awareness

In Nyanza district, data revealed the significant influence parents have on adolescents’ attitudes and behaviors concerning relationships, sexual activity, and reproductive health (Table 4). A substantial proportion of respondents reported discussing reproductive health with their parents, with 44.1% engaging daily and 29.4% weekly, and 58.8% indicating a very open relationship with their parents. Additionally, 97.1% of respondents stated that parental involvement raised their awareness of adolescent pregnancy risks, and 94.1% learned about contraception options. A majority (79.4%) reported receiving parental guidance on responsible sexual behavior. Despite these positive findings, gaps in parental involvement exist; 23.5% described communication with parents as only ‘somewhat open’, while 8.8% reported closed communication. Interviews with community leaders highlighted the challenges parents face in maintaining open dialogues with adolescents, especially around sensitive topics, often due to cultural discomfort.

**Table 4.** Parental communication and its impact on adolescent sexual health.

Category	Response	N	%
Frequency of reproductive health discussions	Daily	225	44.1
	Weekly	150	29.4
	Monthly	90	17.7
	Rarely	45	8.8
Openness in communication on reproductive health	Very open	300	58.8
	Somewhat open	120	23.5
	Closed	45	8.8
	Somewhat closed	30	5.9
	Neutral	15	2.9
Provision of comprehensive reproductive health information	Yes	390	76.5
	No	120	23.5
Comfort level in discussing reproductive health	Always comfortable	270	52.9
	Sometimes	150	29.4
	Never	45	8.8
	Rarely	30	5.9
Parental attitudes towards adolescent pregnancy	Strongly against	390	76.5
	Somewhat against	60	11.8
	Neutral	30	5.9
	Strongly in favor	30	5.9
Perceived importance of parental role in preventing pregnancy	Very important	405	79.4
	Somewhat important	75	14.7
	Not very important	15	2.9
	Neutral	15	2.9
Influence of parental views on relationships and sexual activity	Strongly influence	315	61.8
	Somewhat influence	120	23.5
	Neutral	45	8.8
	Do not influence at all	15	2.9
	Somewhat do not influence	15	2.9
Guidance on responsible sexual behavior	Yes	405	79.4
	No	105	20.6
Impact of parental guidance on understanding pregnancy risks	Increased awareness of risks	495	97.1
	Knowledge of contraception	480	94.1
	Encouraged responsibility	465	91.2
	No impact	15	2.9

Source: This study.

### Support services for pregnant adolescents

The findings in Table 5 highlight the importance of comprehensive support services for adolescent mothers, closely tied to SDGs, Rwandan national initiatives, and international adolescent health standards. Data show that 88.3% of adolescent mothers who received support services during pregnancy found them adequate, suggesting alignment with SDG 3’s aim for healthy lives for all, including maternity healthcare. Conversely, 11.7% reported insufficient support, highlighting disparities that could affect adolescent mothers’ well-being. A strong demand was recorded for academic assistance (65%) and flexible class schedules (86.7%), supporting SDG 4’s goal of inclusive education. Qualitative insights from FGDs further emphasized the need for counseling services that address both mental health and educational support, with participants frequently expressing the desire for a safe space to share their experiences and seek guidance.

**Table 5.** Support services and guidance for pregnant students.

Category	Response	N	%
Received support services during pregnancy	Yes	795	88.3
	No	105	11.7
Adequacy of support services	Yes	615	68.3
	No	285	31.7
Additional support services for pregnant students	Access to prenatal care resources	855	95.0
	Flexible class scheduling	780	86.7
	Counseling services	615	68.3
	Academic accommodation	585	65.0
Parental guidance on responsible sexual behavior	Yes	405	79.4
Impact of parental guidance on pregnancy awareness	Increased awareness of risks	495	97.1
	Knowledge of contraception	480	94.1
	Encouraged responsibility	465	91.2

Source: This study.

Parental engagement also emerged as a major influence, with 97.1% of adolescents indicating that parental guidance raised awareness of pregnancy risks, underscoring the family’s role in fostering healthy sexual behaviors. Despite the availability of some support services, gaps remain, particularly in school-based adjustments (65%) and counseling services (68.3%). Table 5 underscores the need for strengthened institutional support systems to assist adolescent mothers in making informed reproductive health decisions.

## Discussion

This study aimed to assess the factors influencing adolescent pregnancy among adolescents in rural Rwanda and its impact on education, health, and socio-economic outcomes. Findings reveal that limited parental involvement, socioeconomic challenges, and peer influence are key drivers, while access to support services and reproductive resources remains limited.

The findings of the present study underscore the necessity of focused interventions addressing peer influences on adolescent sexual behavior. High levels of peer pressure regarding relationships and sexuality suggest a need for programs that equip adolescents with skills to navigate these dynamics [17]. The data indicating that most respondents experienced their first sexual activity before 18 highlights the urgency of implementing comprehensive sexual education programs, aligning with the goals of adolescent sexual and reproductive health and rights policy, to mitigate early pregnancies and encourage informed decision-making [7]. Despite high awareness levels, limited access to contraception remains a key barrier, and addressing this gap is essential to reducing adolescent pregnancy rates and achieving gender equality. Comfort levels in utilizing reproductive health services also point to an opportunity for enhancing service delivery environments to make them more adolescent-friendly [18].

The significant representation of single-parent and nuclear households emphasizes the potential impact of family involvement on adolescent reproductive health. Findings suggest that strengthening family-based approaches within reproductive health frameworks could enhance education and support for adolescents [19]. Additionally, the cyclical connection between poverty and adolescent pregnancy leads to heightened socioeconomic instability and restricted prospects, contributing to greater rates of maternal mortality and mental health issues among adolescent mothers, consistent with recent studies [20,21].

The stigma surrounding adolescent motherhood exacerbates social exclusion and mental health challenges, aligning with global efforts to reduce discrimination and promote social inclusion. This societal perception negatively impacts self-esteem and limits adolescent mothers’ reintegration into education and the workforce [22,23]. The lack of awareness of support services suggests a need for more extensive information efforts to improve adolescent mothers’ knowledge and access to education, consistent with recent findings [24,25]. Globally, initiatives providing financial literacy and vocational training have shown success in fostering financial independence for adolescent mothers, while support services like childcare aid their pursuit of educational and career goals [26]. The emphasis on counseling and support groups also underscores the importance of psychosocial support for mental well-being [27].

While national strategies exist, the lack of local initiatives reveals significant gaps in community-based support. Research suggests that community-driven programs for adolescent mothers are associated with better long-term outcomes [28]. These findings advocate for a coordinated response across education, healthcare, and community support to foster an empowering environment for adolescent mothers. The detrimental effects of adolescent pregnancy on adolescent mothers’ educational achievements further emphasize the importance of accessible, high-quality education for marginalized populations, aligning with SDG 4 on quality education [29]. The high rate of missed school days due to pregnancy-related issues reflects ongoing educational barriers. While Rwanda has taken steps toward adolescent reproductive health, adolescent mothers’ continued access to education relies on adequate healthcare and financial support [30]. The strong link between economic security and educational retention underscores the need for multidimensional support systems [31]. Although awareness of adolescent pregnancy consequences is high, the gap between awareness and practical support indicates a need for more robust support systems to enable adolescent mothers to continue their education and improve future prospects [32].

The findings also underscore the importance of parental involvement in promoting adolescent reproductive health, aligning with Rwanda’s national reproductive health policy [7] and international commitments made at the International Conference on Population and Development [33]. Parental guidance, reflected in family-based sexual education, fosters responsible sexual decision-making and reduces adolescent sexual risks, contributing to broader goals of gender equality and empowerment. Communication gaps, however, indicate a need for enhanced community and government programs to support parents in discussing sexual health with adolescents. Parental involvement’s impact on adolescents’ understanding of pregnancy risks and contraceptive methods underscores the family’s influence on responsible sexual behavior, which aligns with the convention on the rights of the child and other global standards [34]. The findings advocate for investment in healthcare, educational support, and family-based education, supporting Rwanda’s policies and global SDG commitments. Addressing these gaps could propel Rwanda closer to achieving gender equality, quality education, and improved health outcomes for adolescent mothers, enhancing their prospects for a sustainable future.

## Limitations

This study has several limitations. The capacity to prove a link between adolescent pregnancy and its contributing factors is limited by the cross-sectional design in the quantitative component. Furthermore, the accuracy of responses may have been impacted by social desirability bias, which may have affected self-reported data on delicate subjects like the usage of contraceptives. Additionally, although there was a large sample size, it might not have captured all the details across various geographical areas or socioeconomic backgrounds, especially for marginalized inhabitants. These limitations should be taken into account when planning future studies on adolescent reproductive health in rural Rwanda.

## Conclusion and future perspectives

The support networks for pregnant adolescents are severely lacking, according to this study, especially in the areas of family advice, school-based adjustments, and healthcare access. This study highlighted gaps and the need for structured interventions to address adolescent mothers’ needs. Although many regions have programs offering flexible education alternatives and prenatal care, many adolescent mothers still lack access to counselling services and academic flexibility. These results point to more significant difficulties in coordinating policies with the goals of quality education, good health, and well-being. In addition, family engagement plays a critical role in reproductive health education in a variety of cultural contexts by influencing adolescents’ conceptions of contraception, pregnancy prevention, and the value of lifelong learning. This study highlights the need for more comprehensive and integrated programs that address both health and education to support adolescent mothers in realizing their full potential, particularly as the focus of the world moves towards achieving gender equality and minimizing adolescent pregnancy. By meeting the study’s aim to assess factors influencing adolescent pregnancy and support mechanisms, we underscore how targeted interventions can align with these global objectives.

Subsequent studies ought to concentrate on assessing school-integrated and community-based initiatives that provide comprehensive assistance to adolescent mothers, with an emphasis on marginalized groups and developing areas. To improve service accessibility, especially in remote and underserved areas, new approaches like telemedicine and digital learning platforms should be investigated. Increasing the number of research conducted on the socioeconomic disparities that adolescent mothers face across national borders will shed light on how specific policies might address these issues and lead to more inclusive and successful interventions. Increasing global cooperation amongst families, schools, and healthcare providers will be crucial to establishing a more encouraging atmosphere for adolescent mothers. By filling in these gaps, the international community can help the world reach its development goals, enabling pregnant adolescents to continue their schooling and enhancing their long-term health and financial prospects.
